# The Promise of Whole Genome Pathogen Sequencing for the Molecular Epidemiology of Emerging Aquaculture Pathogens

**DOI:** 10.3389/fmicb.2017.00121

**Published:** 2017-02-03

**Authors:** Sion C. Bayliss, David W. Verner-Jeffreys, Kerry L. Bartie, David M. Aanensen, Samuel K. Sheppard, Alexandra Adams, Edward J. Feil

**Affiliations:** ^1^The Milner Centre for Evolution, Department of Biology and Biochemistry, University of BathBath, UK; ^2^Cefas Weymouth LaboratoryWeymouth, UK; ^3^Institute of Aquaculture, University of StirlingStirling, UK; ^4^Department of Infectious Disease Epidemiology, School of Public Health, Imperial College LondonLondon, UK; ^5^The Centre for Genomic Pathogen Surveillance, Wellcome Genome CampusCambridge, UK

**Keywords:** aquaculture, genomics, whole genome sequencing, molecular epidemiology, infectious disease, pathogens, bacteria, viruses

## Abstract

Aquaculture is the fastest growing food-producing sector, and the sustainability of this industry is critical both for global food security and economic welfare. The management of infectious disease represents a key challenge. Here, we discuss the opportunities afforded by whole genome sequencing of bacterial and viral pathogens of aquaculture to mitigate disease emergence and spread. We outline, by way of comparison, how sequencing technology is transforming the molecular epidemiology of pathogens of public health importance, emphasizing the importance of community-oriented databases and analysis tools.

## The Global Expansion of Intensive Aquaculture

Aquaculture broadly refers to the farming of aquatic organisms, including finfish, shellfish, crustaceans and plants, primarily for human consumption, and has been practized since antiquity. Multiple ancient civilisations independently developed aquaculture systems; 4000 year old bas reliefs show Egyptians fishing for tilapia in artificial ponds, a number of freshwater species were farmed and perhaps domesticated in ancient China and other parts of Asia, ancient Greeks and Romans farmed bivalves and fish in freshwater and saltwater ponds and pre-Columbian systems of weirs in the Amazon are thought to have been constructed for fish cultivation (Costa-Pierce, 2008). However, it was not until the mid-20th century that intensive, industrial-scale aquaculture became viable, and recent decades have witnessed a dramatic expansion such that it is currently the fastest growing food producing sector globally. This expansion has been driven by stresses on terrestrial systems owing to population increase and scarcity of resources, combined with a plateauing of production from capture fisheries world-wide (Garcia and Rosenberg, 2010).

In a world where more than 800 million people continue to suffer from chronic malnourishment (FAO, 2015), and where the global population is expected to grow by another 2 billion to reach 9.6 billion people by 2050, with a concentration in coastal urban areas (UN, 2015), aquaculture plays an increasingly critical role in global food security. In 2013, finfish, molluscs, and crustaceans represented about 17% of the animal protein supply and 6.5% of all protein for human consumption. Finfish alone provided more than 3.1 billion people with almost 20% of their animal protein (FAO, 2014). A milestone was reached in 2014, when for the first time, aquaculture accounted for the majority of fish consumed globally (FAO, 2014). Four decades ago, aquaculture accounted for only 7% of all fish consumed globally, with the vast majority being produced by the capture sector. This increase in aquaculture production has easily outpaced population growth; *per capita* fish supply in 2014 reached a global average of 20 kg, double the global average in the 1960s, with the gap narrowing between developed and developing nations (FAO, 2016). Much of this growth has occurred in Asia, with China alone accounting for 60% of global production, with other major producers being India, Vietnam, Bangladesh, Thailand, and Egypt (FAO, 2016). Ninety-four *per cent* of the 18 million people employed in fish farming in 2014 were Asian, and of the top 10 aquaculture countries, in terms of production, only Norway is classified as high income. Thus the increasing economic reliance on aquaculture is heavily skewed toward developing nations. Total aquaculture animal production in 2014 amounted to 73.8 million tons, worth approximately US$160.2 billion, of which finfish accounted for around 50 million tons (US$100 billion) (FAO, 2016).

The intensification of aquaculture production (the so-called “Blue Revolution”) has some parallels to the agricultural “Green Revolution.” However, there is one important difference. Whereas in agriculture there are a limited number of domesticated species, the dramatic increase in scale of aquaculture production has been accompanied by a commensurate diversification of target species. In 2014 a total of 580 species were farmed around the world, including 362 finfishes, 104 molluscs, and 62 crustaceans (FAO, 2016). Moreover, secondary species are now also being cultivated to improve production. For example, cleaner fish, such as wrasse and lumpfish, are now being farmed as biological control agents to manage salmon lice infestation (Leclercq et al., 2014). This diversification in target species has been accompanied by a very wide range of different farming techniques and husbandry regimes. Farms can be based on fresh, brackish or salt water within cages, earth ponds, tanks, open sea, rafts or raceways, in mono-or mixed culture, from tropical climates to the sub-Arctic (Baluyut, 1989).

Maintaining the steep trajectory of global aquaculture production requires addressing a critical trade-off between intensification and sustainability. The challenges to overcome are many and various, and include: (i) increasingly severe competition with other resource (land/water/feed) users, (ii) deteriorating quality of water supplies resulting from aquatic pollution, (iii) successful integration of aquaculture with other farming activities, and (iv) improvements in environmental management including reduction of environmental impacts and avoidance of risks to biodiversity. However, arguably the biggest threat arising as a consequence of the intensification and globalization of the aquaculture is from infectious disease.

## Emergent Infectious Disease in Aquaculture

Non-communicable disease or sporadic endemic infections have been described in aquatic animals for centuries. However, the development of large-scale intensive aquaculture has been associated with a dramatic increase in severe disease outbreaks caused by a diverse range of pathogens, including parasites, viruses and bacteria (Yin, 2004; Crane and Hyatt, 2011; Woo et al., 2011). These disease outbreaks can be catastrophic for the industry. The Chilean Infectious Salmon Anemia (ISA) outbreak in 2007 bankrupted the industry and left debts of US$1.8 billion (Alvial et al., 2012). Low income rural communities were particularly badly affected, and an estimated 13,000 jobs were lost. Shellfish can be just as badly affected as finfish. Highly virulent shrimp viral diseases have arisen as a result of selection pressure in shrimp farming, subsequently disseminated via transfer of broodstock and larvae (Flegel, 2012). Bacterial diseases can be just as devastating. Between 2010 and 2014 acute hepatopancreatic necrosis disease (AHPND) was estimated to have caused a US$1 billion annual loss to the shrimp farming industry (FAO, 2013). The impact of the disease varied between countries, although an extreme example would be the drop in Thailand’s share of the global shrimp market from 40% in 2011 to 10% in 2012. This disease, originally named early mortality syndrome (EMS), was first identified in China in 2009 and spread successively to Vietnam, Malaysia, Thailand and Mexico by 2013 (Tran et al., 2013; Joshi et al., 2014; Nunan et al., 2014; Soto-Rodriguez et al., 2015). In 2015 the putative causative agent of APHND was identified as a strain, or strains, of the bacterium *Vibrio parahaemolyticus* which had acquired a toxin producing plasmid (Lee et al., 2015). In the intervening period between emergence of the disease and identification of the pathogen APHND had spread almost globally to nations engaged in shrimp aquaculture (FAO, 2013).

Understanding the conditions underpinning the emergence and spread of infectious disease is thus a major challenge for developing a sustainable global aquaculture industry. Pathogen emergence can result through the evolution of novel strains or the international spread and expansion of previously characterized pathogens. New strains causing a novel pathology can potentially emerge through host-switching as a result of intensive mixed farming (Marcos-López and Gale, 2010; Marcos-López et al., 2010), or alternatively new pathogen strains might evolve from a background of commensal organisms. This can occur as a consequence of mutation or the horizontal acquisition of virulence genes via recombination between previously isolated pathogen populations (Wijegoonawardane et al., 2009). Emergent pathogens that have spread rapidly on international scales, termed ‘transboundary’ pathogens, proliferate either through trade of broodstock, fry, fingerlings or mature animals, or as a response to changing environmental conditions. There also are grounds for arguing that certain husbandry practices, including those designed to mitigate the impact of infectious disease, might inadvertently be driving the evolution of increasingly virulent strains (Kennedy et al., 2016). Key husbandry factors impacting on the spread of existing disease, and the emergence of new infectious agents, include stocking densities, compression of the rearing cycle, the use of chemotherapy and a reduction in host genetic diversity (Kennedy et al., 2016). Mixed farms, in which more than one species are farmed together, can increase the potential for pathogen host switches, which is often associated with heightened virulence in the new host. A recent study on the bacterial pathogen *Flavobacterium columnare*, a freshwater pathogen of rainbow trout, indicated that a subset of virulent and competitive strains were being selected in the farm environment (Pulkkinen et al., 2010; Sundberg et al., 2016).

Climate change is also likely to play an increasing role in the emergence of infectious disease in the aquatic environment. Vezzulli et al. (2016) have recently shown a concordance between rising sea surface temperature (SST), the abundance of *Vibrio* bacteria associated with plankton samples and the incidence of human infection caused by these bacteria. In aquaculture settings, the outcome of exposure to fish pathogens is often critically dependent upon both water quality, in particular the concentration of dissolved oxygen, and water temperature. Water temperature impacts both on the replication rate of the pathogen and, being poikilothermic animals, on the immune response of the fish. Whilst some bacterial pathogens, such as *Flavobacterium psychrophilum*, are more virulent at cold temperatures (10°C) most, including *Lactococcus garvieae*, are much more virulent above 15°C. Salmonids are particularly vulnerable to relatively narrow change in temperature with respect to disease susceptibility. This temperature sensitivity has potentially grave implications for disease emergence and spread within the context of a changing climate (Marcos-López and Gale, 2010).

Intensive aquaculture also has the potential to increase the infection load in wild stocks through pathogen ‘spillover’ (Power and Mitchell, 2004). Although it is usually very difficult to gage the severity of this threat, farming of species outside of their natural geographical range is likely to increase the risks due to the introduction of exotic pathogens into susceptible host populations. A controversial example is that of the impact of sea lice spillover from farmed salmon to wild stocks, with some studies pointing to a correlation between lice abundance on farms and the productivity of wild stocks (Krkosek et al., 2011) whilst other studies suggest that spillover of lice alone does not explain increased mortality of natural populations (Marty et al., 2010). The impact and occurrence of pathogen spillover will vary between pathogens, farming systems and local environment. Whilst disease spillover may not impact directly on aquaculture production the negative impact on public perception and the downstream impact on the valuation of farmed aquatic animals should not be underestimated (Hansen and Onozaka, 2011).

Although the precise drivers for infectious disease emergence in aquaculture settings typically remain unclear, it is evident in many cases that strains vary in their virulence and that specific pathogen variants have adapted to the aquaculture settings. This observation has many parallels in agriculture, where certain strains predominate within intensive farming environments, and also in public health where strains of human pathogens appear adapted to the healthcare setting by virtue of multidrug resistance. However, the degree to which disease emergence is accompanied by strong selection imposed by the farmed environment, compared to ‘opportunistic’ infections from environmental reservoirs, depends upon the specific ecology, disease dynamics and etiology.

We discuss some examples of pathogenic Gram-negative (**Box 1**), Gram-positive (**Box 2**) bacteria and viruses (**Box 3**) that are of key importance to aquaculture, with a focus on evidence from molecular typing data. These examples discussed largely affect finfish, as there is typically less detailed evidence available for shellfish pathogens, although we have included a recent study on *Vibrio aestuarianus*, an emerging pathogen of oysters (Goudenège et al., 2015).

**Box 1. Gram-negative bacteria.**

Many different Gram-negative genera are known to cause disease in finfish and shellfish, including *Aeromonas, Edwardsiella, Flavobacterium, Francisella, Photobacterium, Piscirickettsia, Pseudomonas, Tenacibaculum, Vibrio, Weisella*, and *Yersinia* (Austin and Austin, 2012). Disease monitoring and management is often complicated by the ability of bacterial pathogens to persist in the water column, or to asymptomatically colonize farmed species as a constituent of the ‘healthy’ microbiome. Here, we focus on the molecular epidemiology of four exemplar species: *Y. ruckeri, F. psychrophilum, A. salmonicida*, and *V. aestuarianus*. The former three species cause disease within stressed, high density populations of farmed finfish, whilst *V. aestuarianus* causes a serious disease of oysters. Management strategies tend to focus on both mitigating the spread of disease through trade and other human activities, and upon understanding and avoiding the conditions that trigger the transition from a commensal lifestyle to serious pathogen.

***Yersinia ruckeri*** is a rod-shaped bacterium, endemic in cool waters, and is the etiological agent of enteric redmouth disease (ERM). This pathogen affects many finfish species, particularly salmonids, and causes considerable economic losses worldwide. It has been recovered from faces, water and sewage sludge (Willumsen, 1989) and is known to readily form biofilms in the aquatic environment, which helps to account for recurrent infections in trout farms (Coquet et al., 2002). The disease may have been exported on multiple occasions from the USA via the movement of rainbow trout eggs and fry (Austin and Austin, 2012), and an isolate from Europe was first described in 1981 (Lesel et al., 1983). Whilst it is thus possible that the spread of the disease was as a direct consequence of trade and the expansion of aquaculture, it is also possible that *Y. ruckeri* is ubiquitous in aquatic environments worldwide and that local outbreaks result from endemic strains adapting the farm environment (Willumsen, 1989).

Some of the earliest fish vaccines targeted *Y. ruckeri*. Disease in rainbow trout is largely caused by serotype O1 (serovar 1) (Kumar et al., 2015). These strains are related to the motile O1 ’Hagerman’ strains, dating back to the first isolation of *Y. ruckeri* in the 1950s in the Hagerman valley, Idaho, and which have had widespread use in immersion vaccination (Wheeler et al., 2009; Barnes, 2011). The dominance of this clone as a source of infection in intensively farmed rainbow trout is suggestive of either specific adaptations to the host or to the conditions associated with the farm environment.

Although the O1 vaccine was initially very effective, vaccine failures in rainbow trout were reported from the early 1990s (Austin et al., 2003). Welch et al. (2011) characterized a variant serotype 01 strain, biotype 2 (BT2) that was causing outbreaks worldwide even in vaccinated fish. The key distinguishing phenotypes of BT2, which are likely to be linked to the ability to escape the vaccine, are the loss of motility due to the absence of peritrichous flagella and a lack of secreted phospholipase. These phenotypes arise from a very small set of mutations that were found to have occurred independently on at least four occasions.

Early work suggested that the virulence of the *Y. ruckeri* serogroup O1 BT2 clone in trout is linked to an enhanced ability to survive in serum (Davies, 1991b). It has since been shown that the O1 serotype is specifically virulent in rainbow trout, whereas Atlantic salmon are typically more susceptible to infection by *Y. ruckeri*, and a greater diversity of strains are virulent in this host. This was recently confirmed by the phenotypic characterization of 109 *Y. ruckeri* isolates from Atlantic salmon and 26 isolates from rainbow trout (Ormsby et al., 2016). Whereas the majority of the strains isolated from rainbow trout corresponded to the common serogroup O1 BT2 clone, the Atlantic salmon isolates corresponded to 19 different clones. However, all the Atlantic salmon strains in this study corresponded to BT1. A separate study observed that BT2 has arisen independently in Atlantic salmon in Tasmania (Barnes et al., 2016). In this study *Y. ruckeri* isolates were sequenced from a collection that spanned 27 years, covering the entire period of time over which the salmonid farming industry has become established in Tasmania. BT2 was only observed intermittently between 2004 and 2007 and has not been detected since. This may be due in part to the ongoing reduction in motility of BT1 that infected salmon during this time period, which may have allowed BT1 to maintain a selective advantage over BT2. In addition to O-antigen serotyping (Romalde et al., 1993), *Y. ruckeri* populations have been characterized using outer membrane protein (OMP) analysis (Davies, 1991a) and more recently using MLST (Bastardo et al., 2012, 2015). These data shed light on the global biogeography of this species, as well as providing inferences of recent and ancient demographic history. More specifically, the MLST data provided evidence three distinct clades representing isolates from North America, South America, and Europe. The population structure reflects a complex combination of ancient range expansion and more recent migration due to human activity (Bastardo et al., 2015).

***Aeromonas salmonicida*** subsp. *salmonicida* is a non-motile psychrophilic bacteria that causes furunculosis, so called due to the boils (or furuncles) that develop on the skin or musculature typical of chronic disease in older fish. The disease can spread either through direct contact with infected fish or via contaminated water. The acute form of the disease, which more commonly affects younger fish, leads to rapid septicaemia and death within 2–3 days (Dallaire-Dufresne et al., 2014). This is one of the most costly and well-studied diseases of salmonids, particularly Atlantic salmon, and affects both farmed and wild stocks (Lillehaug et al., 2003). Reports of furunculosis epidemics in wild trout in North America and Europe date back to the late 19th century, with major epidemics in wild stocks in the UK and Denmark during the 20th century (Bakke and Harris, 1998). Outbreaks in farmed marine salmon in Scotland and Norway began in the mid-1980s. Although the spread of the disease has been mitigated by the deployment of vaccines since the 1990s, and have been particularly successful for Atlantic salmon, these vaccines are expensive and can cause serious side effects.

*A. salmonicida* subsp. salmonicida is one of five recognized *A. salmonicida* sub-species. However, this scheme is far from comprehensive, and any strains identified as *A. salmonicida* but which do not fall within these sub-species are loosely assigned as “atypical.” Atypical strains can also cause ulcerative disease in salmonids and non-salmonids including cod, halibut, wolffish (Dallaire-Dufresne et al., 2014). In particular, atypical *A. salmonicida* can cause furunculosis in wrasse and lumpsuckers (Collins et al., 1991). These ‘cleaner fish’ are of increasing economic importance due their use as biological control agents for salmon lice infestation and efforts are currently underway both in Scotland and Norway to develop robust farming methods for these species.

*A. salmonicida* possess an impressive armory of virulence factors, including proteases, lipases and other extracellular molecules as well as a number of toxins (effectors) secreted directly in the host cytoplasm via a plasmid-encoded type three secretion system which is essential for infection in fish. The *A. salmonicida* cell is also covered in a tetragonal protein (encoded by the *vapA* gene) which associates with lipopolysaccharide to form a hydrophobic “A-layer” that protects the cell from macrophages and is responsible for autoagglutination. Gulla et al. (2016) have recently characterized the *vapA* gene from a large collection of typical and atypical *A. salmonicida*. They noted 14 discrete *vapA* sequence clusters that correspond to the known sub-species but also, to a large degree, to different host species, thus pointing to a need for species specific vaccines based on different A-layer types. Although multilocus sequence analysis (MLSA) has been used to study the taxonomy and diversity within the *Aeromonas* genus (Roger et al., 2012; Colston et al., 2014), MLST has not been specifically applied to *A. salmonicida* epidemiology.

***Flavobacterium psychrophilum*** is a psychrotrophic species and the causative agent of rainbow trout fry syndrome (RTFS) and bacterial cold water disease (BCWD) in salmonids (Austin and Austin, 2012). Rainbow trout and coho salmon are particularly susceptible to the disease, and extensive losses have also been reported in farmed and wild ayu (*Plecoglossus altivelis altivelis*) (Wakabayashi et al., 1991). No broad spectrum vaccine is available, and disease management requires close surveillance and the use of antimicrobials. *F. psychrophilum* is endemic in the environment, and can transmit both horizontally and probably also vertically. As with *Y. ruckeri* and other bacterial pathogens, a key question relating to the epidemiology of this species is the contribution of sporadic infection from endemic environmental lineages, compared to the spread via trade of farm-adapted lineages.

Arai et al. (2007) used PFGE to characterize 64 isolates of *F. psychrophilum* from ayu and other fish hosts in Japan, and noted evidence for association between PFGE and host species. Chen et al. (2008) similarly used PFGE to characterise 139 isolates from farmed rainbow trout and wild (spawning) coho salmon mostly from Idaho in the USA. Whilst they noted that samples from both species were characterized by the presence of a small number of predominant PFGE clusters, the isolates from spawning coho salmon were markedly more diverse than those from farmed trout, consistent with transmission within and between farms of specific clones that may have adapted to the farm environment.

An MLST scheme for this pathogen was proposed in 2008 using 11 loci and based on the full genome sequence of the reference strain JIP02-86, which revealed very high rates of recombination (Nicolas et al., 2008). A subsequent MLST study on isolates from Swiss fish farms was published, and these data did not confirm host associated with specific clonal groups (Strepparava et al., 2013). Nilsen et al. (2014) reported a very large MLST study of 560 isolates from four Nordic countries; Norway, Finland, Denmark, and Sweden. This diverse isolate collection was recovered from different hosts and environmental sources between 1983 and 2012. Although the population as a whole was diverse, representing 81 sequence types (STs) and 12 clonal complexes (CC’s; clusters of closely related STs), the data confirmed the presence of a dominant clone, ST2 (CC10), which represented 65% of all the isolates. This confirmed other MLST studies using the same scheme that also reported the predominance of ST2 in France (Siekoula-Nguedia et al., 2012) and Switzerland (Strepparava et al., 2013). In Norway this clone is particularly monomorphic, suggesting a recent introduction into this country. ST2 is strongly associated with rainbow trout, and appears to have disseminated rapidly between rainbow trout farms due to some selective advantage, possibly decreased sensitivity to quinolones (Shah et al., 2012). Isolates from other salmonid hosts and from the environment are more diverse and freely recombining. The combination of a freely recombining, diverse ‘background’ population and a small number of predominant widespread clones conforms to an ‘epidemic’ population structure as originally proposed for the human pathogen *Neisseria meninigitidis* (Smith et al., 1993).

***Vibrio aestuarianus*** has recently been causing commercially damaging disease in farmed oyster stocks, particularly in France. Goudenège et al. (2015) used whole genome sequencing to characterize 14 *V. aestuarianus* strains isolated both before and during the French outbreak, which began in 2012. They noted two distinct clades, but as both clades contained isolates that pre-date the outbreak there was no evidence of the emergence of a single virulent clade that could explain the increase in oyster mortalities. However, this study demonstrated a key role in virulence of a single regulatory gene, *varS.* This gene, a recognized virulence factor in other Gram-negative bacteria, encodes a signal transduction histidine-protein kinase, which regulates the expression of a secreted metalloprotease. The authors noted that a *varS* had undergone a frameshift in one non-virulent strain, leading to reduced expression of the metalloprotease. The authors went on to provide experimental evidence that the *varS* gene is indeed an important virulence factor of *V. aestuarianus* in oysters. This study provides an excellent example of how whole genome sequencing, combined with experimental data, can provide powerful evidence concerning virulence and pathogenicity in shellfish pathogens.

**Box 2. Gram-positive bacteria.**

The frequency of reports of aquaculture diseases caused by Gram-positive pathogens has been steadily rising globally over the last 20 years or so. There are three main Gram positive taxa of relevance to aquaculture disease management, the two firmicute genera *Lactococcus* and *Streptococcus*, and *Renibacterium salmoninarum*, a member of the *Micrococcaceae*. Below we will give an overview of two key species, *L. garvieae* and *S. iniae.* These are both very diverse ecological generalists that can cause disease in a wide range of host species including, on occasion, humans. *R. salmoninarum*, the causative agent of bacterial kidney disease in salmonids, was the first fish pathogen to which whole genome sequencing was applied for molecular epidemiology (Brynildsrud et al., 2014), and this study is discussed in the main text.

***Lactococcus garvieae*** is a highly diverse species that causes a fatal hemorrhagic septicaemia called lactococcosis that can result in a loss of 50% of total production. This pathogen causes disease in fish species worldwide, particularly rainbow trout but also a host of other wild and farmed fishes including Japanese yellowtail (Kusuda et al., 1991), gray mullet (Chen et al., 2002), Japanese eel (Kusuda et al., 1991), Red Sea wrasse (Colorni et al., 2003), bottlenose dolphin (Evans et al., 2006), tilapia (Evans et al., 2009), and flounder (Baeck et al., 2006). *L. garvieae* has been also been isolated from farmed giant freshwater prawns (*Macrobrachium rosenbergii*) (Chen et al., 2001). It is also a causative agent of bovine mastitis, and is associated with dairy and meat products, vegetables and cereals, and occasionally poultry (Ferrario et al., 2012). A few cases of human infection have been described, thus it is recognized as an emerging pathogen in public health, and its ability to survive at relatively low temperatures compared to typical human pathogens can help explain the recent isolation of this organism as contaminating agent in blood products prior to transfusion (Kozakai et al., 2016).

Although a “streptococcal” disease was first recorded in rainbow trout in Japan in the 1950s, the causative organism was not described until the 1983, when it was assigned as *Streptococcus garvieae* but reclassified as *L. garvieae* shortly afterward in Schleifer et al. (1985). The type strain of *S. garvieae* (ATCC 43921) was an isolate recovered from a case of bovine mastitis in the UK; it is perhaps unsurprising that no link was made at this time between a pathogen taken from a mastitic udder in the UK and disease outbreaks in rainbow trout in Japan decades earlier. This connection remained unnoticed even when large outbreaks of lactococcosis started to occur in rainbow trout farms in southern Spain from 1988 (Palacios et al., 1993), with the causative organisms being assigned as *Enterococcus* sp. However, the link between fish disease and *L. garvieae* was made in 1991 when archived samples taken from infected fish in Japan dating back to the 1970s were characterized (Kusuda et al., 1991). Although initially erroneously re-assigned as *Enterococcus seriolicida*, subsequent studies finally reclassified *E. seriolicida* as a junior synonym of *L. garvieae* (Doménech et al., 1993).

Subsequent to the initial outbreaks in southern Spain, the disease spread rapidly throughout Europe and elsewhere in the world. Outbreaks associated with high mortality and severe economic losses have been reported in trout farms worldwide, including in Australia, South Africa, Turkey, the USA (Nelson et al., 2016) and Iran (Sharifiyazdi et al., 2010). Within Europe, there was a rapid spread between rainbow trout farms amongst Mediterranean countries particularly in Italy, Portugal and Balkan countries, and further north into France and the UK. The disease was probably transmitted directly through the movement of infected fish, and indirectly (horizontally) through water (Eyngor et al., 2004). *L. garvieae* is now endemic throughout much of continental Europe and the UK.

The appearance of disease symptoms is highly temperature sensitive, with outbreaks on farms most commonly occurring during the summer months when water temperatures exceed 16°C. It is thus likely that increasing summer temperatures resulting from climate change are at least partly responsible for the northward spread of this pathogen within Europe (Marcos-López et al., 2010). The critical role of temperature in clinical outcome was demonstrated by Algöet et al. (2009) who tested the virulence of a *L. garvieae* strains in rainbow trout by intraperitoneal injection and noted substantially lower mortality at 14°C (3%) than at 16°C (28%) or 18°C (67%).

Initial typing studies based on restriction fragment length polymorphism (RFLP) ribotyping were suggestive of a link between Italian outbreak isolates and the early Japanese isolates, consistent with transmission from Japan into southern Europe (Eldar et al., 1999). However, this link was far from certain as the Japanese isolates were mostly recovered from marine fish and showed serological differences to the isolates from European trout. In fact PFGE data subsequently revealed the Italian-Japanese strains to be quite distinct from the Spanish outbreak isolates and from a French isolate (Vela et al., 2000). The hypothesis that that there was a single introduction into Europe from Japan, followed by regional spread, can therefore be discounted. A more extensive RFLP based ribotyping study revealed clonal regional spread within endemic southern regions, but a more diverse mixture of strains from France where only sporadic infections were reported (Eyngor et al., 2004). The data also revealed that the majority of the isolates within the *L. garvieae* population corresponded to one of two major lineages, with one group corresponding to serotype 1 isolates from Israel and France, and the other serotype 2 isolates from Spain, Greece, and Bulgaria. PFGE data also revealed the presence of two distinct types within Spain, and further confirmed extensive heterogeneity and multiple sources of infection (Vela et al., 2000).

The existence of two distinct and major lineages of *L. garvieae* was later confirmed using multilocus restriction typing (MLRT) (Ferrario et al., 2012) and MLST (Ferrario et al., 2013). These studies characterized isolates form a broad range of ecological sources, including infected fish, raw milk, vegetables, humans, and poultry but, interestingly, found no evidence of concordance between the two major lineages and ecological origin.

***Streptococcus iniae*** is responsible for outbreaks of septicaemia and meningitis (streptococcosis) in at least 27 species of cultured and wild freshwater and marine finfish, including trout, tilapia, bass, yellowtail and flounder (Romalde et al., 1993; Agnew and Barnes, 2007). The name of this species reflects the host from which it was first recovered, *Inia geoffrensis* (a freshwater dolphin). It is associated with warm water, particularly in North America, Asia-Pacific (including Australia) and the Middle East, and it is relatively well-studied partly due to its ability to cause invasive disease in humans through the handling of infected fish. Serotype specific vaccines (including bacterins, attenuated vaccines and DNA vaccines) have generally proved successful, though mutations in the capsular region have been shown underlying vaccine escapes through serotype switching (Zhang et al., 2014; Heath et al., 2016). Molecular typing using ribotyping, random amplification of polymorphic DNA (RAPDs) and rep-PCR have been useful in ruling out putative epidemiological links, for example between North America and Israel (Eldar et al., 1997), and it is likely that local disease is mostly caused by established endemic strains that have gone undetected until relatively recently. Whilst PFGE studies have revealed that isolates recovered from human disease are relatively clonal, isolates recovered from farmed and wild fish are highly diverse, with multiple genotypes being detected within and between different farms. A number of virulence factors in *S. iniae* have been characterized, many of which are similar to those found in group A streptococci (GAS) (Baiano and Barnes, 2009; Zhang et al., 2014).

**Box 3. Viruses.**

A number of serious viral aquaculture diseases are notifiable, and are very difficult to treat or vaccinate against (OIE, 2016). However, molecular epidemiological studies have made significant contributions to understanding, and thus managing, the emergence and spread of viral diseases (Snow, 2011). Key aquaculture viruses include infectious salmon anemia virus (ISAV), infectious pancreatic necrosis virus (IPNV), viral hemorrhagic septicemia virus (VHSV), infectious hematopoietic necrosis virus (IHNV), Channel catfish virus (CCV) and Cyprinid herpesvirus 3 (CyHV-3), previously known as Koi herpesvirus (KHV). There are also a range of viral diseases that cause significant problems for farmed shellfish species. These include white spot syndrome virus, yellow head virus genotype 1 and Taura syndrome (OIE, 2016). As has already been discussed with respect to ISA, these viruses can cause devastating outbreaks with severe commercial consequences. Below we discuss CyHV-3 and VHS.

**Cyprinid herpesvirus 3**, CyHV-3 (previously known as Koi herpesvirus; KHV) is the causative agent of a lethal, highly contagious and notifiable disease of carp and ornamental koi (*Cyprinus carpio*). In addition to being farmed for angling and for exhibitions, carp is also one of most intensively cultivated fish globally for human consumption. As is the case with other aquaculture pathogens, clinical outcome following exposure to CyHV-3 is critically dependent upon water temperature and the disease is highly seasonal. CyHV-3 has spread rapidly to at least 33 countries since it was first observed in Israel, USA and Germany on the late 1990s, probably mostly as a result of global trade and koi exhibitions. As with other members of genus *Cyprinivirus*, it is phylogenetically distinct, and has become a focus of fundamental research into host-virus interactions (Rakus et al., 2013). Full genome sequencing of three isolates from the USA, Israel, and Japan revealed an unusually large genome (295 Kb) consisting of a large central region flanked by inverted 22 Kb inverted repeats each encompassing eight ORFs (Aoki et al., 2007). The presence of loss-of-function mutations in genes predicted to encode membrane glycoproteins has been speculated to be associated with host switching and disease emergence in the carp host from an environmental reservoir population. Although the three genome sequences revealed little genetic diversity, it is clear that variant from Japan is distinct from those isolated from the USA and Israel, which are more similar to each other. Subsequent studies revealed two sub-lineages in Asia, and at least seven within the European lineage. VNTR genotyping has been used successfully to define a new ‘intermediate’ lineage from Indonesia, and to demonstrate the coexistence of different strains within a single location, which is consistent with multiple introduction events and rapid transmission (Avarre et al., 2011).

**Viral Hemorrhagic Septicaemia** (VHS) is a highly infectious disease of freshwater and marine fish. In contrast to CyHV-3, where infections are typically asymptomatic at temperatures below 16°C, symptoms of VHS are rarely noted in temperatures above 15°C (Oidtmann et al., 2013). The causative agent, viral hemorrhagic septicaemia virus (VHSV) is an World Organisation for Animal Health listed pathogen (OIE, 2016), and management of the disease following detection may involve destruction of fish on affected premises, implementation of movement controls and extensive surveillance prior to resumption of normal trading. The genome is 11-Kb (containing six genes), and it is a single-stranded RNA virus belonging to the genus *Novirhabdovirus*, as does IHNV. Four distinct genotypes have been identified that appear to be associated with different geographical regions, environments (freshwater or marine) and host species (Snow et al., 1999). Genotypes I, II, and III are associated with European waters (with sub-lineages also showing geographical restriction (Abbadi et al., 2016), whereas genogroup IV is typically recovered from North America (Snow et al., 2004). Genotype I has been divided into five subtypes (Einer-Jensen et al., 2004) and genotype IV into three subtypes. VHS has caused substantial die-offs in the Laurentian Great Lakes (Stepien et al., 2015). A recent study of VHSV and IHNV in Italy based on the sequence of the glycoprotein (Abbadi et al., 2016) suggested that there had been multiple introductions of VHSV into Italy.

## The Promise and Challenges of Whole Genome Sequence Data for Molecular Epidemiology

Molecular epidemiology encompasses a synthesis between molecular biology, typing, evolutionary biology, population biology and epidemiology (Galvani, 2003). As discussed below, whole genome sequencing represents the ‘ultimate’ typing methodology in terms of discriminatory power, and the advantages and opportunities for infectious disease management afforded by the advances in sequencing technology are numerous and profound. Moreover, much of the methodology developed for pathogens that infect humans, including bioinformatic pipelines, data archiving and management and statistical approaches to analyzing the data, are directly applicable to aquaculture settings.

Before discussing in more detail the significant benefits from adopting whole genome pathogen sequencing for aquaculture disease management, there are two key caveats to be highlighted. First, it is critical to appreciate that any molecular typing dataset, including whole genome sequencing, is always most productively considered alongside rich metadata pertaining to the epidemiological source and clinical phenotypes of the isolates. Sequence data alone will rarely inform on novel disease management strategies. Second, the contribution sequencing technology will make toward developing a sustainable future for the global aquaculture industry is ultimately dependant on the degree to which these data, and associated metadata, can be freely archived, accessed, and interrogated. As discussed below, the technical ability, and political will, to share resources and combine data from international sources is critical for efficient global surveillance, for providing early warnings of new emerging pathogens, and for fully exploiting the data for novel diagnostics and vaccine development. There are, of course, considerable challenges with respect to agreeing common standards and resolving issues relating to data ownership and commercial sensitivities.

Molecular typing methodologies have long been deployed to understand and monitor the emergence and spread of bacterial pathogens of public health and veterinary relevance. A key advance in the late 1990s was the development of multilocus sequence typing (MLST) which combined advances in automated sequencing technology with the rapid expansion of the internet (Maiden et al., 1998; Pérez-Losada et al., 2013). The ability to access data via a website provided the means, for the first time, for near real-time molecular epidemiological surveillance on a global scale (Urwin and Maiden, 2003). Crucially, the establishment of MLST for major pathogenic species was accompanied by the development of freely accessible web-based databases incorporating generic and intuitive software tools^1^. These databases grew rapidly as MLST became more widely adopted, thus further increasing the utility of the scheme, and demonstrating the power of community-oriented resources (Brehony et al., 2007). Due to the limitations of sequence technology in the late 1990s, MLST was designed to assay only a tiny proportion of genomic variation (typically, seven genes). This means MLST data can be insufficiently discriminating to distinguish localized disease outbreaks from cases of endemic disease, or to reconstruct the global or national spread of individual clones (Pérez-Losada et al., 2013; Habib et al., 2014).

The development of next-generation sequencing (NGS) platforms, and in particular the Illumina MiSeq, made it possible to generate data for the vast majority of genes within a bacterial genome from large population samples, thus revealing the *minutiae* of genetic change genome-wide. The MLST databases held at pubmlst.org were thus modified and expanded to encompass data for potentially 1000s of genes rather than seven (Sheppard et al., 2012; Maiden et al., 2013). The ability to assay variation in 100s, or even 1000s, of genes revealed layers of hitherto hidden sub-variants within single ‘strains’ (or MLST genotypes), and often these sub-variants are geographically restricted (Baker et al., 2010; Harris et al., 2010). The unprecedented discriminatory power of whole genome sequencing has been applied to both human and animal pathogens to reconstruct transmission pathways within outbreaks, track the source of foodborne pathogens (Dearlove et al., 2016; Ronholm et al., 2016) and infer microevolutionary changes within a single host, over time-scales of weeks or months (Falush, 2009; Azarian et al., 2016; Lillebaek et al., 2016). Patterns of pathogen transmission have been inferred across all epidemiological scales; within single hospital outbreaks (Senn et al., 2016; Willems et al., 2016), animal hosts (Sheppard et al., 2013; Mather et al., 2016), hospitals or farms (Chang et al., 2016; Kamath et al., 2016; Reuter et al., 2016), or across countries or continents (Aanensen et al., 2016).

Of course, as with MLST data (Maiden, 2006), a pre-requisite for using genome sequencing for studying the epidemiology and/or evolution of bacterial pathogens is the initial assembly of a representative population sample. Whilst studies on local or geographically restricted outbreaks necessarily require deep sampling from within the locales affected, a broad phylogenetic context can often prove invaluable for inferring when, and from where, any given outbreak may have arisen. Phylogeography is a general term that combines the spatial dynamics of the pathogen with patterns of molecular evolution, and thus describes the distribution of specific variants over space. Bayesian statistical procedures, as implemented in BEAST (Drummond and Rambaut, 2007), can be used for reconstructing the emergence of a new virulent or resistant pathogen, and its subsequent phylogeographic spread (Holden et al., 2013). Similarly, estimates of mutation rate, which can also be made using models implemented BEAST (Drummond and Suchard, 2010), allow the reconstruction of time calibrated phylogenies, and thus the possibility of linking specific evolutionary changes, such as the emergence of new lineages (or the extinction of old ones), with demographic changes resulting from perturbations in the selective landscape. Such perturbations may either result from specific disease management strategies, such as the deployment of vaccines or the administration of a new antibiotic, or broader environmental challenges such as those resulting from climate change. Importantly, just as phylogeographic studies require samples representing, as far as possible, the spatial range of the study population, so the accuracy of mutation rate estimates similarly depends on the temporal spread of the sample. As a rule of thumb, this typically means the inclusion of at least a small number of bacterial strains archived a minimum of 10 years, or ideally 20 years, prior to the contemporary population.

A good example of how these approaches have helped further our understand of the evolution and epidemiology of pathogens important for public health is the study by Holden et al. (2013). The authors sequenced almost 200 genomes of an epidemic clone of Methicillin resistant *Staphylococcus aureus* (EMRSA-15) from multiple countries in Europe and, importantly, included isolates dating back over 20 years. Using the Bayesian approaches discussed above, they inferred that this clone emerged in the English West Midlands in the mid-1980s. This inference was strongly supported by a second, independent, line of evidence: an important characteristic of this clone is resistance to flouroquinolone and, strikingly, the time and date of clonal emergence inferred from the sequence data coincided almost perfectly with the first clinical trials of this drug in the UK. In addition to public health, WGS studies on livestock pathogens have provided insights into the selection pressures resulting from intensive food production processes, and how these processes can increase or limit opportunities for pathogen transmission (Franz et al., 2014). WGS data also underpins an increased understanding of fundamental micro-evolutionary processes in bacterial pathogens, such as homologous recombination, gene gain and loss, genetic drift, and the strength and direction of selection (Castillo-Ramírez et al., 2011; Yahara et al., 2016).

**FIGURE 1:**
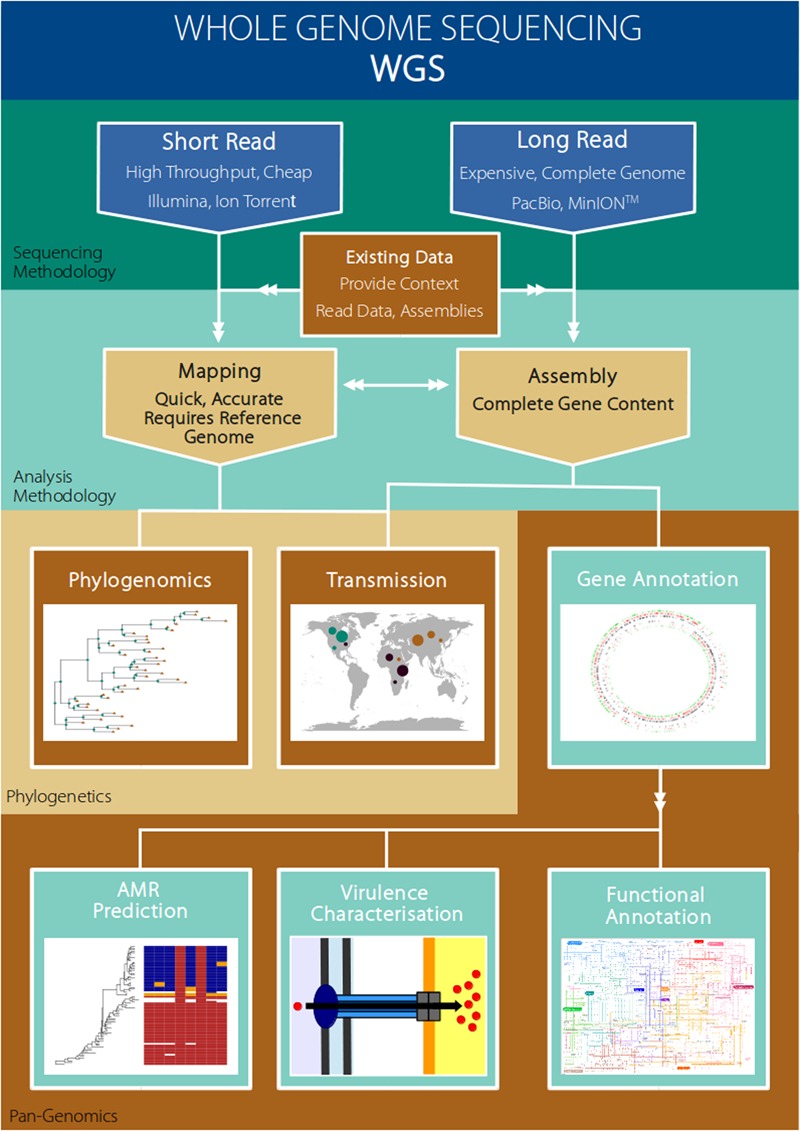
**Example workflow of how whole genome sequencing of pathogens maybe used to address research areas of particular relevance to the aquaculture setting**.

Although the ability to assign an isolate to one type or another using traditional typing methodologies, such as MLST or pulsed-field gel electrophoresis (PFGE), may indirectly provide phenotypic information (e.g., virulence or resistance profile), this is reliant on these traits having already been associated (linked) with the given genotype. In contrast, as WGS data provides a complete catalog of gene content, as well as SNPs (single nucleotide polymorphisms) and indels (insertions and deletions), it is possible to directly mine the data for virulence or resistance genes in order to predict these phenotypes and inform on optimum management options. In particular, mobile genetic elements (MGEs) such as phage, plasmids or genomic islands commonly carry genes conferring enhanced virulence or resistance to antibiotics (Gordon et al., 2014; Lindsay, 2014; Walker et al., 2015). Alternatively, these MGEs may carry genes underpinning adaptation to specific hosts, ecological niches or environmental pressures (Sheppard et al., 2013; Holt et al., 2015; Hsu et al., 2015). Rapid advances are being made in the development of sophisticated statistical procedures, or genome-wide association studies (GWAS) approaches, to identify genes or single SNPs that are associated with key phenotypic traits such as virulence, resistance, or host adaptation (Farhat et al., 2014; Howell et al., 2014; Skallerup et al., 2015; Falush, 2016; Laabei and Massey, 2016). The broad utility of WGS data thus lies in simultaneously tracking the emergence and spread of a given variant, and also in providing predictive information concerning key phenotypic traits (Feil, 2015). **Figure 1** presents some examples of how whole genome sequencing may be applied to benefit major areas of research that are relevant to pathogen evolution and epidemiology in aquaculture (**Figure 1**).

## The Utility of Whole Genome Sequencing within Aquaculture Settings

Many of the questions and examples above have clear and direct relevance for aquaculture settings. It is, in principle, equally feasible and valid to use WGS to track disease emergence and spread within and between individual fish-farms as it is within and between hospitals. Of particular relevance to aquaculture is the ability to identify and track specialized strains that have become adapted to specific hosts, such as *Aeromonas salmonicida* (**Box 1**) or viral hemorrhagic septicemia virus (**Box 3**). For example, recent outbreaks of VHSV have occurred in farmed wrasse used to control salmon lice in Scotland and Iceland (Munro et al., 2015). The strains that cause disease in the wrasse were shown to be genogroup III, but these strains do not cause overt disease symptoms in Atlantic salmon (Munro et al., 2015; Matejusova et al., 2016). This is especially relevant for the World Organisation for Animal Health notifiable pathogen species, the presence of which may incur the destruction of stock or a temporary ban on trading between states (OIE, 2016).

Whilst important efforts are being made to incorporate and modify risk-based methods, and other statistical and modeling approaches, from terrestrial to aquaculture systems, these endeavors are often constrained by a lack of basic data concerning pathogen movement, host and environmental adaptations and population dynamics (Murray and Peeler, 2005; Oidtmann et al., 2013). However, given the long and highly successful history of the development and use of robust molecular epidemiological tools in aquaculture (Boxes 1–3) there can be little doubt that whole genome sequencing is set transform the field (Snow, 2011). There are already a large number of complete reference genomes for a range of aquaculture associated pathogens (**Table 1**). These represent a strong foundation to utilize NGS for molecular epidemiology in aquaculture. Phylogeographic analysis, namely the ability to infer geographical structuring and patterns of transmission by distinguishing between almost identical variants of a single pathogenic strain, informs on many of the challenges underpinning the management of aquaculture diseases. These tools are of particular relevance for the identification of transmission routes between aquaculture producing countries or regions, and may help inform novel disease management strategies. More specifically, such approaches can be used to unequivocally demonstrate whether the disease emergence in a specific region has resulted from transmission of the pathogen from elsewhere, or has originated from within the ‘native’ endemic microbial ecosystem. An excellent recent example, and a superb “roadmap” for how WGS might be applied to aquaculture pathogens more broadly, is the recent study by Barnes et al. (2016) (**Box 1**). These authors sequenced 51 genomes of *Yersinia ruckeri*, an important pathogen of salmonids, from Tasmania (*n* = 44) and New Zealand (*n* = 7) and analyzed these alongside six genomes from other countries already in the public domain. The temporal range of the isolates was 27 years. These data demonstrated that the disease-causing strains in Australia and New Zealand are distinct from those in the Northern Hemisphere, and are instead local endemic strains.

**Table 1:** WGS studies of selected bacterial and viral aquaculture pathogens.

	Pathogenic species	Reference
**Bacterial**	*Aeromonas salmonicida* subsp. *salmonicida*	Reith et al., 2008; Charette et al., 2012; Valdes et al., 2015; Vincent et al., 2015, 2016
	*Aeromonas salmonicida* subsp. *achromogenes*	Han et al., 2013
	*Francisella noatunensis* subsp. *orientalis*	Sridhar et al., 2012; Figueiredo et al., 2016; Gonçalves et al., 2016
	*Flavobacterium psychrophilum*	Duchaud et al., 2007; Wu et al., 2015; Castillo et al., 2016; Neiger et al., 2016
	*Lactococcus garvieae*	Aguado-Urda et al., 2011; Morita et al., 2011; Ricci et al., 2012
	*Piscirickettsia salmonis*	Eppinger et al., 2013; Pulgar et al., 2015
	*Renibacterium salmoninarum*	Wiens et al., 2008; Brynildsrud et al., 2014
	*Streptococcus agalactiae*	Delannoy et al., 2012; Liu et al., 2013; Pereira Ude et al., 2013; Kayansamruaj et al., 2015
	*Streptococcus iniae*	El Aamri et al., 2013; Pridgeon et al., 2014; Zhang et al., 2014
	*Vibrio anguillarum*	Naka et al., 2011; Li et al., 2013; Busschaert et al., 2015; Holm et al., 2015
	*Vibrio harveyi*	Kondo et al., 2015
	*Vibrio parahaemolyticus*	Yang et al., 2014; Letchumanan et al., 2016
	*Yersinia ruckeri*	Nelson et al., 2015; Wang et al., 2015; Barnes et al., 2016; Liu et al., 2016
	*Vibrio aestuarianus*	Goudenège et al., 2015
	*Aeromonas hydrophila*	Rasmussen-Ivey et al., 2016
**Viral**	Cyprinid herpesvirus (CyHV-3)/Koi herpesvirus (KHV)	Aoki et al., 2007; Hammoumi et al., 2016
	Infectious hematopoietic necrosis virus (IHNV)	Jia et al., 2014; Wang et al., 2016
	Infectious pancreatic necrosis virus (IPNV)	Dadar et al., 2014
	Infectious salmon anemia virus (ISAV)	Markussen et al., 2008; Cottet et al., 2010; Mérour et al., 2011
	Taura syndrome virus (TSV)	Mari et al., 2002
	Viral hemorrhagic septicemia virus (VHSV)	Schütze et al., 1999; Kim et al., 2013; Ito et al., 2016; Schönherz et al., 2016
	White spot syndrome virus (WSSV)/white spot bacilliform virus (WSBV)	Yang et al., 2001; Rodriguez-Anaya et al., 2016
	Yellow head nidovirus (YHV)	Sittidilokratna et al., 2008

Each specific setting and pathogen will, of course, pose particular questions. We outline below further recent key studies that illustrate how whole genome sequencing has already begun to transform the field.

*Renibacterium salmoninarum* is the causative agent of bacterial kidney disease in salmonids, and a successful Multilocus VNTR (variable number of tandem repeats) Analysis (MLVA) scheme has also been developed (Matejusova et al., 2013). *R. salmoninarum* was also the first aquaculture pathogen for which WGS was used to reconstruct patterns of international transmission and microevolutionary events (Brynildsrud et al., 2014). These data are consistent with the hypothesis that this pathogen was introduced into Europe from North America over the last 50 years, possibly via the trade in eggs. The study also provided proof-of-principle that WGS can be used to detect outbreak variants within a single fish-farm or neighboring farms. The recovery of essentially indistinguishable isolates from the same geographical location, but from different host species, provided strong evidence for frequent host-switching within the farm environment, which has implications for both the management of farmed aquatic animals and also in gaging the risk of spillover into wild stocks. These data also revealed a deep phylogenetic division within the population, possibly reflecting historical allopatry between Pacific and Atlantic wild stocks.

Whole genome sequencing of aquaculture pathogens, including both viruses and bacteria, has also been central to the development of novel diagnostic tools and bespoke typing methodologies. For example, Avarre et al. (2011) noted that a large proportion of the variation present in the genomes of CyHV-3 is restricted to a small number of hyper-variable tandem repeat loci, from which they developed a discriminatory and relatively cheap and easy VNTR scheme (variable number of tandem repeats). A more recent study showed that it is possible to sequence CyHV-3 genomes directly from tissues of infected carp, which revealed high levels of mixed infection (Hammoumi et al., 2016). Whole genome sequencing is also paving the way to the development of new vaccine targets through the identification of novel surface expressed proteins (Barnes and Silayeva, 2016) and reverse vaccinology (Andreoni et al., 2016). Seth-Smith et al. (2016) used whole genome sequencing to characterize two novel intracellular pathogens of gilthead seabream. Many other studies are now using whole genome sequencing to confirm inferences from other typing methods. For example cases of mixed infection of *Streptococcus agalactiae* and *Francisella noatunensis* subsp. *orientalis* in farmed Nile Tilapia (Assis et al., 2016), and comparative genomics were used to supplement a comprehensive MLST study of *Aeromonas hydrophila* infection in farmed catfish in the USA (Hossain et al., 2014).

Recent comparative genomics papers have also focused on understanding pathogenicity and the distribution of virulence factors in bacterial populations (Sudheesh et al., 2012). Busschaert et al. (2015) analyzed 15 draft genomes of *Vibrio anguillarum* and noted very few genetic differences between strains with differing virulence properties, indicating that only very small genomic changes might underlie major clinical differences. Castillo et al. (2016) combined six novel genome sequences of *F. psychrophilum* from fish farms with five publicly available sequences to show very few differences in the distribution of virulence factors or phenotypic traits over large geographical distances. Finally, a comparative genomics analysis of a range of *A. salmonicida* sub-species provided a detailed picture on the genome dynamics of this species and provided evidence that insertion sequences may be a driving force in the evolution of a psychrophilic lifestyle (Vincent et al., 2016).

Recent advances in third generation sequencing technologies may also impact on the application of WGS to the aquaculture sector. Long read technologies, such as the Pacific Biosciences’ PacBio, have dramatically reduced the time and cost of producing high-quality draft and complete genome sequences (Zhang et al., 2012). Another innovation in the field of sequencing technologies, the Oxford Nanopore MinION^TM^, has the potential to reduce the cost and time investment necessary to set-up sequencing centers. The MinION^TM^ has been used for ‘in the field’ sequencing of the recent Ebola and Zika virus outbreaks (Faria et al., 2016; Quick et al., 2016). Considering that the majority of aquaculture producing regions are in developing nations this may be a revolutionary development for sustainable disease control strategies that use WGS for molecular tracing. Although the cost of the technology is currently prohibitive, future developments may lead to this technology being a cost-effective alternative in regions without existing sequencing infrastructure. In addition, the turnaround time for the MinION^TM^ from sample to sequence has been shown to be short in comparison to current technologies. When used during the Ebola outbreak, sequences of the virus were often available within 24 h of the sample being submitted.

## The Management and Analysis of WGS Data within a Community-Oriented Framework

The studies cited above illustrate how the generation of WGS data can advance basic bioscience and aquaculture disease management. However, the optimal exploitation of this technology is dependent on a community-oriented infrastructure being in place to facilitate resource sharing. Rapid advances are being made in the development of community-oriented databases and analysis tools for public health pathogens, and these tools are largely transferable to aquaculture pathogens. Freely available databases that store, manipulate and analyze WGS data, include BIGSdb (Maiden et al., 2013), Enterobase^2^, Whole Genome Sequence Analysis^3^, and commercial systems are also available^4^. Other systems deliver rapid annotation such as the RAST server (Aziz et al., 2008), or can predict antibiotic resistance profiles (Zankari et al., 2012; Bradley et al., 2015), or analyze gene content variation (Page et al., 2015). Bioinformatics tools are rapidly becoming more powerful and more accessible to the community, and the development of cloud-based informatics infrastructure provides the means both to provide easy access to these tools and the necessary computing power to analyze very large datasets (e.g., climb.ac.uk).

**FIGURE 2:**
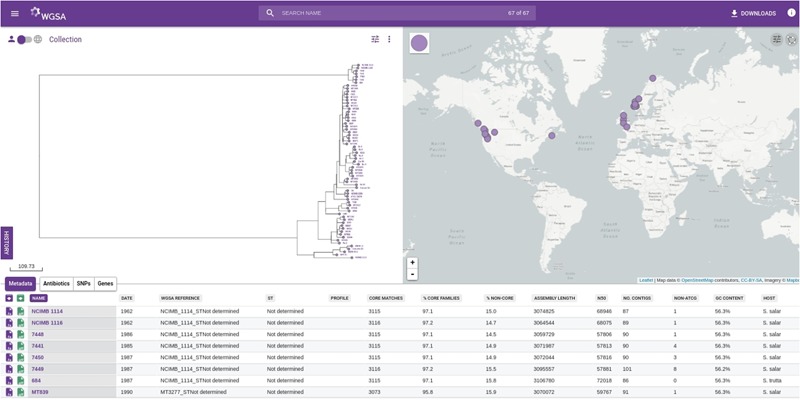
**Screenshot of the WGSA.net whole genome MLST scheme of 68 *Renibacterium salmoninarum* isolates from the collection of Brynildsrud et al. (2014).** The assemblies and metadata are hosted at WGSA.net (http://wgsa.net/rensm/brynildsrud). The left-hand window shows the phylogenetic tree of the isolates built from the core genome MLST scheme. The right-hand window shows the geographical distribution of isolates. The bottom window shows metadata and summary statistics for individual isolates from the collection.

**FIGURE 3:**
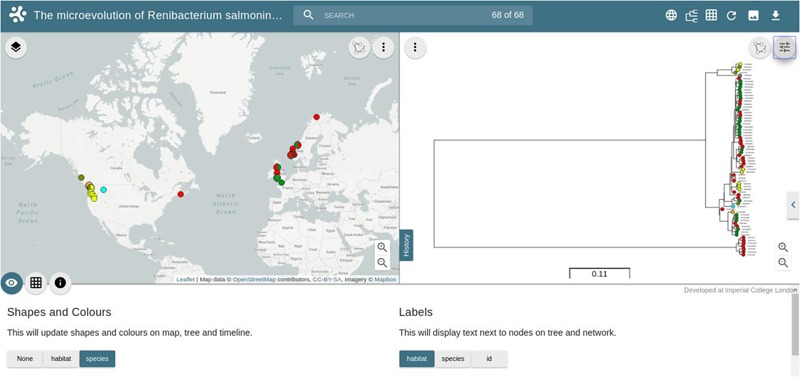
**Screenshot of the Microreact project for 68 *R. salmoninarum* isolates from the collection of Brynildsrud et al. (2014).** The left-hand window shows the geographical distribution of isolates colored by the host species from which they were isolated. The right-hand window shows the consensus phylogenetic tree inferred using a Bayesian Markov Chain Monte Carlo approach, with a generalized time-reversible model (Brynildsrud et al., 2014). The bottom window shows toggle buttons for displaying alternative metadata labels, shapes and colors on the phylogenetic tree and world map.

In order to illustrate the utility of these databases, we have loaded the *R. salmoninarum* whole genome sequence data of Brynildsrud et al. (2014) on to WGSA.net. Additionally, we have used the same dataset to generate a core genome MLST scheme (http://wgsa.net/rensm/brynildsrud; **Figure 2**). WGSA.net is a web application for uploading, processing, clustering and exploration of microbial genome assemblies in real time. Genome assemblies and metadata files can be added simply through “drag and drop” onto a web browser. The system then provides a suite of analysis options including quality metrics for the data, reconstruction of MLST profiles (if a scheme has been pre-defined), resistance (or virulence) prediction, ‘core’ gene set identification, gene content differences, and phylogenetic analysis, SNPs or genes specific to a given clade (useful for developing novel diagnostics) are provided and data are displayed interactively via trees and maps showing the geographical sources of the isolates. The dataset of Brynildsrud et al. (2014) has been included as a public collection, allowing for other research groups to integrate their own public or private data to provide context to WGS data of local or regional outbreaks of *R. salmoninarum*. Simpler tools have also been developed for visualizing and exploring open-access datasets. Full genome phylogenies of Ebola and Zika viruses, and patterns of genomic variability, are freely available to explore at nextstrain.org/ebola and nextstrain.org/zika respectively. The Microreact web-server (microreact.org) is designed to allow sharing and visualization of metadata and phylogenetic trees and serves as an open platform for linking the outputs of whole genome analysis pipelines to epidemiological and other metadata for collective interpretation (Argimón et al., 2016). We have utilized this tool to visualize the *R. salmoninarum* data of Brynildsrud et al. (2014) (https://microreact.org/project/N1KdygLt; **Figure 3**). This system provides a number of powerful features, such as the ability to select isolates based on geographical location (directly from the map), phylogenetic position (directly from the tree) or time of isolation.

## Conclusion: The Future of Pathogen Genomics in Aquaculture

It is clear that whole genome sequencing is set to revolutionize the molecular epidemiology of aquaculture pathogens as it has for those pathogens of relevance to public health. Reconstructing pathogen transmission routes on local and global scales will form an important part of this, but the specific challenges relating to aquaculture disease management are far more multilayered and complex than for public health. These challenges encompass the biology and ecology of both the pathogen and the host (including host adaptation, and different modes of transmission), trading patterns, husbandry and food production, as well as monitoring pathogen emergence and spread due to shifting environmental pressures, in particular climate change. Whole genome sequencing will make important contributions to all these aspects, and others besides. However, in order to capture the full potential of these data, it is critical to develop common standards and pipelines, and open, community-oriented database infrastructures and analysis tools that can be tailored toward these specific questions.

An important first step will be the generation of a set of reference datasets for key pathogens, both bacterial and viral, which represent wide geographical, temporal and phenotypic diversity which can be readily accessed and interrogated. To this end, we are in the process of expanding the dataset of *R. salmoninarum* to include isolates from Chile, and generating datasets for other key aquaculture pathogens including *F. psychrophilum, Piscirickettsia salmonis, V. anguillarum, Y. ruckeri, L. garvieae* and CyHV-3. This has been made possible in part by the WGS-aqua project (wgs-aqua.net), funded under the UK Research Councils (BBSRC/NERC) sustainable aquaculture call, with important contributions from Cefas. These data will be stored in the standard read and assembly archives but also made available through WGSA.net. These high quality, open-access reference datasets will provide a valuable tool for the aquaculture community and that will showcase the utility of WGS data for understanding the emergence, transmission, and molecular epidemiology of aquaculture pathogens. Furthermore, we hope that they also highlight the importance of community based, community supported and open-access databases for monitoring and management of disease through this and subsequent publications.

If the current trends in the deployment of WGS for disease management for public health are replicated for aquaculture, then we will witness a transition to the routine use of genomics for molecular epidemiology and the generation of datasets containing 100s, if not 1000s, of isolates. Although technical advances in informatics are already providing the means to openly share, analyze and contribute to these datasets, significant barriers relating to data ownership and commercial sensitivities will remain. The consequences of disclosing the presence of a pathogen on a farm when the farm is not legally obliged to do so may clearly be prohibitive. However, we are optimistic that the significant benefit of access to high quality reference collections with broad geographical and temporal coverage will become readily apparent within the aquaculture community. Just as with public health, both academics and governmental agencies should be encouraged to incorporate WGS into routine investigation and, as far as possible, to make the data available to the community.

Presently the application of WGS to bacterial genomes is more cost-effective and less technically demanding than viral or parasite genomes. However, methodological advances in enrichment for viral sequences may shortly make large scale investigation of the epidemiology of viral genomes feasible (Bonsall et al., 2015). Protists and other eukaryotic parasites represent a further frontier that, due to space constraints, have not been discussed at length in this article and which currently pose more serious methodological challenges. However, the rate at which sequencing technology is developing, with the advent of entirely new platforms capable of generating long reads, such as the Oxford Nanopore Minion^TM^, there are grounds to be optimistic that large genomics datasets, representing 1000s of specimens, will soon be within reach even for these more challenging disease agents.

## Author Contributions

All authors contributed significantly to the manuscript, with the initial drafts being prepared by SB, DV-J, and EF.

## Conflict of Interest Statement

The authors declare that the research was conducted in the absence of any commercial or financial relationships that could be construed as a potential conflict of interest.

## Footnotes

1 www.pubmlst.org

2 https://enterobase.warwick.ac.uk/

3 http://WGSA.net

4 http://www.ridom.com/seqsphere
